# Supplementary material for: Mining heterogeneous clinical notes by multi-modal latent topic model
Source: PLoS One. 2021 Apr 8;16(4):e0249622. doi: 10.1371/journal.pone.0249622 (PMC8031429; doi:10.1371/journal.pone.0249622)
Supplement: S1 File — (PDF) [file pone.0249622.s001.pdf]

S1 Learned topics from MIMIC-III notes

Topics learned with the single-note type model and multi-note type model are shown in S1 and S2 Tables respectively.

S2 Measuring The Impact of Pre-processing and Uncertainty

As discussed in Section , our pre-processing procedures include standard practices for text processing and topic models, such as removing stop words, removing punctuations, and removing too frequent or infrequent words. Among all the operations, the design choice that might have a significant impact on our conclusions is the maximal document frequency for defining too frequent words, and therefore we studied the impact of this factor on the performance on the two prediction tasks. Specifically, on each task, we varied the maximal document frequency from 10% to 70% with an interval of 5%, and at each level we ran experiments 10 times with different random seeds for data splits.

S3a to S3B Fig show the average performance and error bar of multi-note model on mortality prediction as measured by AUPRC and AUROC. Similarly, S4a to S4b Fig show the performance on PMV prediction. On both tasks, there is little variation in the performance across different random seeds, and the performance is consistent with varying maximal document frequency, indicating that our model is insensitive to the choice of data processing.

S3 Study of Inference Algorithm Performance

In order to study the performance of our inference algorithm, we ran experiments on simulated datasets with various controlled parameters (such as the number of patients, number of tokens, vocabulary size and the number of note types). We then correlated the topic mixture learned from this simulated data with the ground truth topics and calculated the correlation between the two.

We simulated the data by first sampling D patients and K topics from 2 Dirichlet distributions parameterized by $\alpha = 0.1$ and $\beta_t = 0.1$ respectively where t is a note type. Then for each patient, we sampled a topic from a multinomial distribution over the patient topic mixture and M words from a multinomial distribution over the sampled topic mixture. All the simulated datasets were curated for $K = 50$ topics. For the experiments in this section, we simulated different datasets by varying the number of patients (D), number of tokens (M), vocabulary size and number of note types (t). We also removed from the vocabulary words that occur in less than 5 patient notes as a part of preprocessing.

The results for these experiments are in S5 **Fig.** There are five main experiments

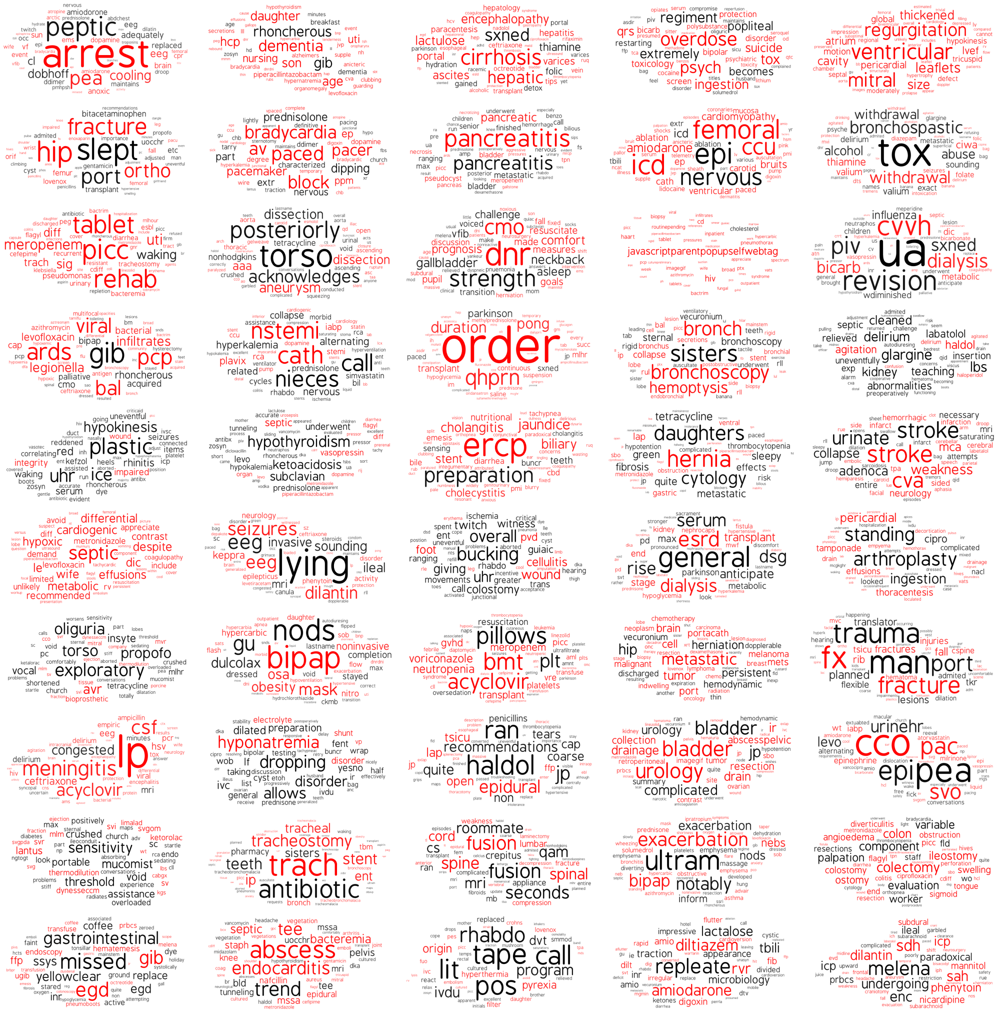

S1 **Fig.** Word clouds of the 50 topics from **single-note** model. Red indicates the words written by physicians and black indicates the words written by nurses.

we ran in this section where we measured the correlation between the learned topics and the ground truth topics as a function of,

1. the number of patients, while keeping the number of tokens in the patient notes and the vocabulary size fixed to 1500 and 2500 respectively. The results are shown in S5a Fig.

2. the number of tokens in each patient's notes, while keeping the number of patients and the vocabulary size fixed to 4000 and 2500 respectively. These results are in S5b Fig.

3. the size of the vocabulary, while keeping the number of patients and number of tokens fixed to 4000 and 1500 respectively. These results are in S5c Fig.

4. the number of different note types, while keeping number of patients, number of tokens and the vocabulary size fixed to 4000, 1500 and 2500 respectively. We choose these parameter values to be close to the real dataset used in the paper. The results are shown in S5d Fig.

We conducted two more experiments where 1) we dropped a note type randomly for each patient and measured the correlation between the learned topics and the ground truth topics and 2) we compared the correlation values between the patient-topic distribution (theta) of our method and a baseline by varying the number of note types. The baseline was an LDA method that considered the same words from different note types as the same word and simply added up their frequencies. For both these experiments, the number of patients, number of tokens and vocabulary size were fixed to 4000, 1500 and 2500. These results are in S5e and S5f Fig.

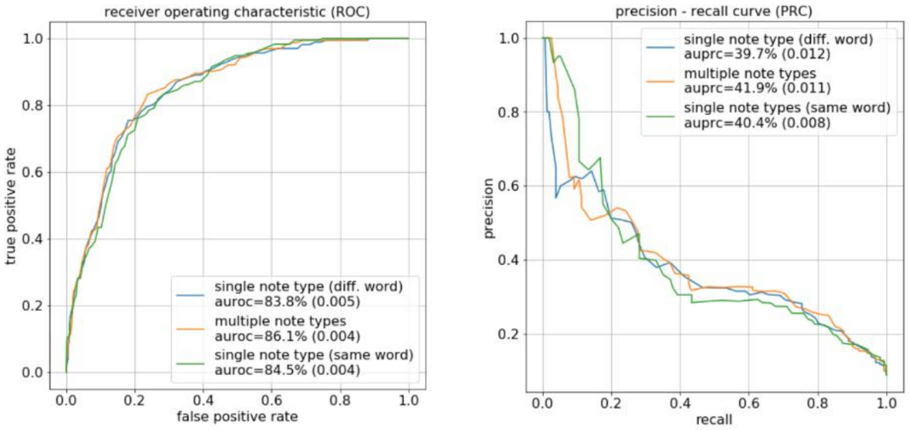

S2 Fig. ROC and Precision-recall curve for binary mortality prediction. We trained the two single-note topic models and the multi-note topic models on the first 48 hours of the clinical notes for each patient. We then trained a separate logistic regression classifier that took the patient-note topic mixture as input and predicted whether the patient is going to stay on MV for more than 7 days. The trained topic models and logistic classifiers were then applied to the test patients to make the predictions of PMV duration. The prediction accuracy was evaluated by ROC and precision-recall curves via 5-fold cross validation. The figure inset shows the AUROC and AUPRC values for each model, and the standard deviations across 10 random splits are in parenthesis.

Topic	Category	Cohesiveness	Disease
0	Cardiology	5	Cardiac arrest
1	Mix	2	
2	Gastroenterology	5	Liver cirrhosis with complications
3	Psychiatry	5	Suicide
4	Cardiovasular	5	Valvular regurgitation
5	Traumatology	4	Hip fracture
6	Cardiology	4	AV block / pacemaker
7	Gastroenterology	4	Pancreatitis
8	Cardiology	3	
9	Substance	4	Alcohol withdrawal
10	Infection	3	
11	Cardiology	4	Aortic dissection / Aneurysm
12	Mix	2	
13	Miscelleneous	1	
14	Nephrology	3	Renal replacement therapy
15	Mix/Gastroenterology/Respiratory/Infection	3	
16	Cardiology	5	Non-ST-elevation myocardial infarction (
17	Mix	2	
18	Respiratory	3	
19	Mix	2	
20	Mix/Neurology	2	
21	Mix/Metabolism	2	
22	Gastroenterology	5	Cholangitis / Cholecystitis
23	Mix	2	
24	Neurology	4	Stroke
25	Mix	2	
26	Neurology	4	Seizure
27	Mix/Infection/Vascular	3	
28	Nephrology	4	End stage renal disease (ESRD)
29	Mix	3	Procedure
30	Mix	2	
31	Respiratory	4	Obstructive sleep apnea (OSA)
32	Hematology	5	Hematology disease & treatment & comp
33	Oncology	4	Metastatic carcinoma
34	Traumatology	4	Fracture
35	Neurology	4	Meningitis
36	Mix/Electrolyte	2	
37	Mix	2	
38	Urology	5	Bladder tumor with complications
39	Cardiology	5	Cardiac arrest
40	Mix/Cardiology	2	
41	Respiratory	4	Tracheostomy
42	Neurology	4	Vertebral fracture
43	Respiratory	4	Chronic obstructive pulmonary disease (C
44	Gastroenterology	4	Diverticulitis / Colectomy
45	Gastroenterology	4	GI bleeding
46	Infection/Cardiology	5	Infectious endocarditis
47	Mix	2	
48	Cardiology	4	atrial fibrillation with rapid ventricular r
49	Mix/Neurology	3	

S1 Table. Learned single-note-type topics from MIMIC-III notes

We observed in all experiments, high correlation scores (higher than 0.9) between the topics recovered by our method and the ground truth, which showed that our inference algorithm is fully capable of recovering latent topics from data.

With increase in the number of patients (number of documents), the correlation values increased which means that the model better learns the ground truth topic distribution. This is expected because there are more samples for the model to learn from. Notably, on a small corpus with only 1000 documents, our inference algorithm can still successfully recover topics (average correlation score higher than 0.92).

With increase in the number of tokens (words per documents), the correlation values also increased. This is also expected because again there are more samples for the model to learn from.

With increase in the vocabulary size, keeping the number of patients and number of tokens fixed, the correlation values decreased as the same number of samples are used to learn the topic distributions over a larger vocabulary. However, we note that the correlation values are still high (greater than 96%).

Similarly, with increase in the number of note-types, in order to keep the overall

Topic	Category	Cohesiveness	Disease
0	Cardiology	5	Cardiac arrest
1	Cardiology	5	STEMI
2	Mix/Cardiology	3	
3	Respiratory	4	Pneumonia
4	Mix	2	
5	Cardiology	5	Valvular regurgitation
6	Orthopedic	5	Vertibral fracture & treatment
7	Infection	4	Multiple organ failure in sepsis
8	Respiratory/Cardiology	4	Pneumonia & atrial fibrillation with rapid ventricular resp
9	Rehabilitation.	4	Bed-ridden
10	Miscellenous	1	
11	Cardiology	4	Emergency medicine
12	Traumatology	5	Fracure
13	Miscellenous	1	
14	Orthopedic	5	Hip/Femoral fracture
15	Hematology	5	Hematology disease & treatment & complication
16	Cardiology	2	
17	Neurology	5	Subarachnoid hemorrhage(SAH)
18	Oncology	5	Metastatic carcinoma
19	Respiratory	5	Chronic obstructive pulmonary disease (COPD) / Obstruct
20	Mix/Cardiology/Respiratory	3	
21	Infection	4	Multiple organ failure in sepsis
22	Gastroenterology	5	Cholangitis / Cholecystitis
23	Surgery	3	
24	Neurology	5	Subdural hemorrhage (SDH)
25	Neurology	5	Delirium
26	Substance	5	Alcohol withdrawal with seizures
27	Cardiology	4	Bradycardia
28	Neurology	5	Meningitis
29	Cardiology	5	Aortic dissection / Aneurysm
30	Gastroenterology	4	Intestinal obstruction
31	Neurology	4	Intracranial hemorrhage (ICH)
32	Cardiology	5	Valvular regurgitation
33	Hematology/Cardiology	5	Cardiac involvement in lymphoma
34	Infection/Vascular	5	Cellulitis
35	Respiratory	5	Pneumonia
36	Gastroenterology	4	Pancreatitis
37	Gastroenterology	5	Liver cirrhosis / Transplant
38	Cardiology	5	Infectious endocarditis
39	Respiratory	4	Hemoptysis / Pneumothorax
40	Surgery/Traumatology	3	
41	Gastroenterology	5	Liver cirrhosis with complications
42	Allergy	5	Allergic reaction / Angioedema
43	Respiratory	4	Tracheostomy
44	Nephrology	5	End stage renal disease (ESRD)
45	Psychiatry	1	Suicide
46	Cardiology	5	Ventricular tachycardia (VT)
47	Gastroenterology	5	GI bleeding
48	Neurology	5	Stroke
49	Cardiopulmonary	4	Pulmonary embolism

S2 Table. Learned multi-note-type topics from MIMIC-III notes

number of tokens fixed, we reduced the number of tokens from each note-type as a result of which, the correlation values decreased. Notably, even with 1500 tokens split in 4 note types, i.e. 375 tokens per note type, our inference algorithm can still successfully recover topics (average correlation score higher than 0.94).

We also tried modifying this experiment to the setting where the number of tokens increases (t times), and observed that the correlation values increased with increase in number of note-types (shown in the plot below). Here we noted that the correlation values for t=1 and t=2 are lower because of the lower number of tokens compared to the vocabulary size. As a result, the model has fewer samples to learn the topic distribution from.

Even when some patients randomly miss 1 of the 2 note-types, the model does a good job of learning the ground truth topics as shown by the high correlation values. In the figure, when t=1, 2, the box plot shows the correlation values between the learned and ground-truth topic distribution for note-types 1 and 2 and respectively. The multi-note model better learns the patient-topic distribution (shown by higher correlation values) compared to the baseline LDA model that treats all note-types as the same.

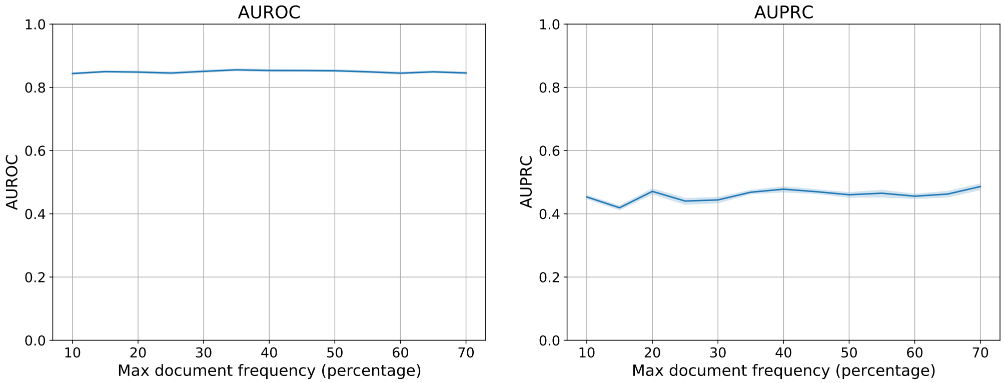

(a) AUROC **(b)** AUPRC

S3 **Fig.** Performance on mortality prediction with varying maximal document frequency.

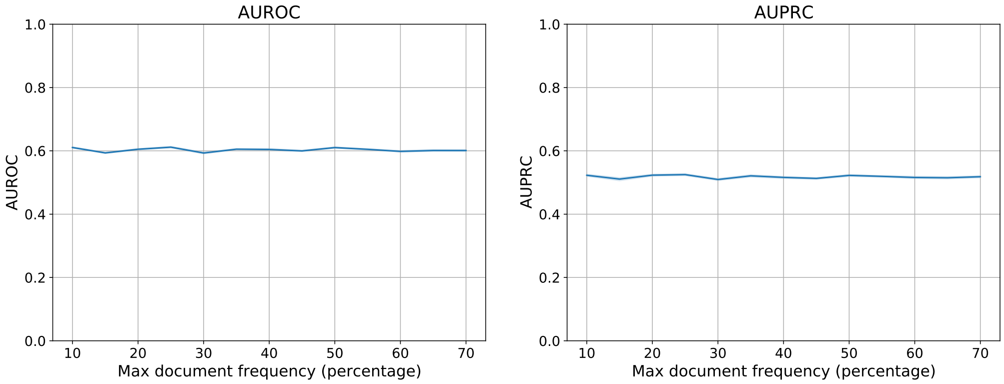

(a) AUROC **(b)** AUPRC

S4 **Fig.** Performance on PMV prediction with varying maximal document frequency.

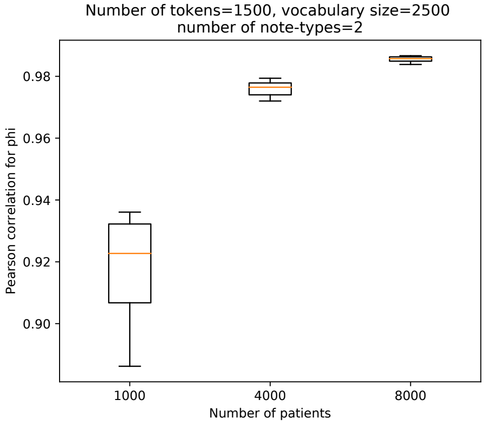

(a) As a function of the number of patients

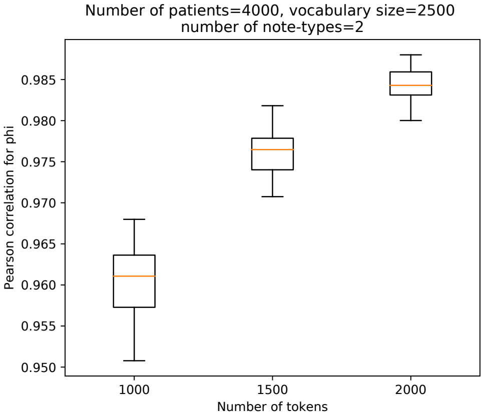

(b) Number of tokens

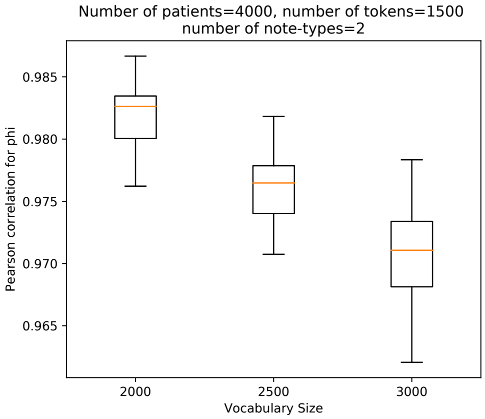

(c) Vocabulary size

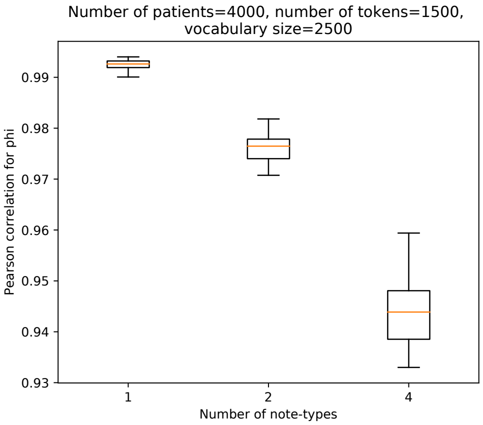

(d) Number of note types

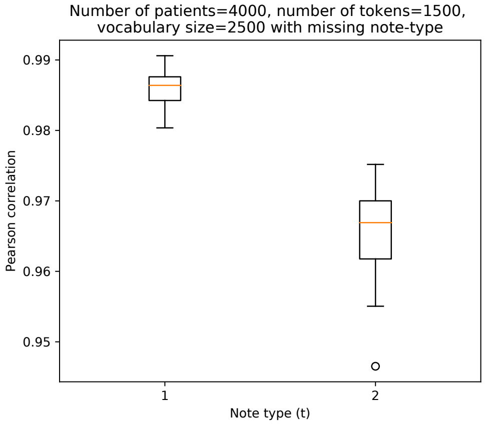

(e) Randomly dropping note types

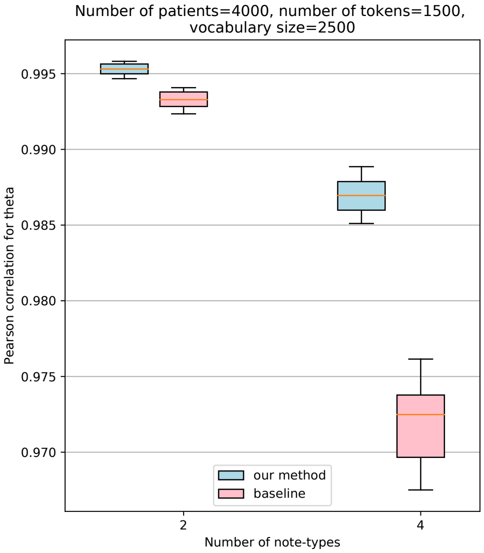

(f) Comparison with baseline

S5 Fig. Results of simulation experiments. Correlation between the topics learned by our model and the ground truth topics for various experimental settings.

S4 Data availability

All relevant data are available on Github:
https://github.com/li-lab-mcgill/heterogeneous_ehr
