# Supplementary material for: Mining heterogeneous clinical notes by multi-modal latent topic model
Source: PLoS One. 2021 Apr 8;16(4):e0249622. doi: 10.1371/journal.pone.0249622 (PMC8031429; doi:10.1371/journal.pone.0249622)
Supplement: Attachment — Submitted filename: Response to Reviewers.pdf [file pone.0249622.s002.pdf]

Reviewer 1

The authors have proposed an interesting study on the text modelling for electronic health records. In particular, the authors conceived that different note types should be modelled differently in a single framework. Therefore, they have proposed the modelling solution. Overall, the idea is well-motivated with good writing styles in a logical manner. In particular, I would like to highlight that the authors did ask for the physicians' involvements in the proposed study. Such experiments reflect the authors' efforts and its clinical relevance. I have the following minor comments:

1. It could be nice if the authors could show other methods for comparisons.

Our response: Thank you for the suggestions. We compared our multi-modal topic model with Latent Dirichlet Allocation (LDA), which is the single note type (same word) version of our model.

2. The time complexity analysis or running time could be provided.

Our response: In theory, the computational complexity of our model is the same as the standard LDA model: O(DKJ). Here D is the number of documents (i.e., patients/admissions), K is the number of topics, and J is the size of the vocabulary (i.e., the total number of distinct EHR codes across all data types). In practice, because the actual EHR data are extremely sparse, only a very small fraction of the EHR codes is observed in any given patient's admission. Also, our model employs collapsed variational inference such that the topic inference operates on the counts of the distinct EHR code for each patient. Therefore, we can formulate the time complexity based on topics and the total number of distinct pairs of patient and EHR-code observed M in the training data: O(MK).

3. The source code could be released.

Our response: We have released the code in the revised version:
https://github.com/li-lab-mcgill/heterogeneous_ehr

Reviewer 2

Summary

I appreciated the opportunity to read this interesting paper. The authors propose an extension of the seminal Latent Dirichlet Allocation topic model of Blei et al. (2003) such that documents (i.e., medical notes) about a given patient written by different authors can be accommodated. This is accomplished by introducing multiple topic-word probability distribution matrices that are author-specific. They describe a variational Bayes estimation procedure to approximate the posterior distribution of the model parameters. They evaluated generalizability (i.e., prediction quality) using cross-validation on a subset of the MIMIC-III

(Johnson et al., 2016). Results suggest marginal or no improvement in prediction quality over two alternative LDA models. Two facets of topic interpretability from the three models were evaluated by two external raters with relevant expertise. Overall, the proposed model is a useful and extensible generalization of LDA that could be useful in a variety of fields and applications beyond medicine.

Major Issues

+ Page 3, 4: All notes for a given patient are assumed to have the same topic proportion vector θ_j. This seems to be a very strong and unrealistic assumption since each note may quite naturally contain different topics or different compositions of topics, a much more plausible scenario that the present model does not accommodate. For example, a nurse may write a note that focuses on a different subset of the K topics than a doctor.

Our response: We would like to clarify that in our study the notes of an admission, instead of a patient, are grouped together as one document and therefore assumed to have one topic composition. While notes written in different admissions might have different focuses on the topics, it is reasonable to assume notes within a single admission have mostly the same topics, including notes written by different professionals (Section 2.2, 86-90).

+ Page 4: The notation in the variational algorithm equations is inconsistent and symbols and subscripts are not always clearly defined. Please carefully define the notation of the different counts n. In particular, Equation (5) introduces new notation that is never defined, specifically Ψ and W_t.

Our response: Thanks for raising this point. We have clarified the notations (Section 2.1, 64-65).

+ While an estimation algorithm for the proposed model is introduced, its performance is not evaluated. Without a simulation study to evaluate the quality of the point estimates obtained from the variational algorithm, it is impossible to know (a) if it can correctly recover the parameters of the proposed model (b) the necessary data requirements (e.g., number of documents, length of documents) and (c) the impact of potential complicating factors on model recovery (e.g., vocabulary size, the number of note types, impact of missing note types for some participants). Many applications of topic models in social science and medicine apply these models to much smaller data sets, particularly, short documents and a small number of documents where the models can break down. It would be valuable for both further methodological development and good practice in application to study the statistical performance of this model systematically.

Our response:

(Section S7 in supplementary material) In order to study the performance of our inference algorithm, we run experiments on simulated datasets with various controlled parameters (such as the number of patients, number of tokens, vocabulary size and the number of note types). We then correlate the topic mixture learned from this simulated data with the ground truth topics and calculate the correlation between the two. If our inference algorithm is

capable of recovering the hidden topics from documents, the recovered topics should strongly correlate with the ground truth.

We simulated the data by first sampling D patients and K topics from 2 Dirichlet distributions parameterized by \alpha = 0.1 and \beta_t=0.1 respectively where t is a note type. Then for each patient, we sampled a topic from a multinomial distribution over the patient topic mixture and M words from a multinomial distribution over the sampled topic mixture. All the simulated datasets were curated for K=50 topics. For the experiments in this section, we simulated different datasets by varying the number of patients (D), number of tokens (M), vocabulary size and number of note types (t).

The results for these experiments are in the figures below. There are five main experiments we ran in this section where we measure the correlation between the learned topics and the ground truth topics as a function of,

1. the number of patients, while keeping the number of tokens in the patient notes and the vocabulary size fixed to 1500 and 2500 respectively.
2. the number of tokens in each patient's notes, while keeping the number of patients and the vocabulary size fixed to 4000 and 2500 respectively.
3. the size of the vocabulary, while keeping the number of patients and number of tokens fixed to 4000 and 1500 respectively.
4. the number of different note types, while keeping the number of patients, number of tokens and the vocabulary size fixed to 4000, 1500 and 2500 respectively.

We observe in all experiments high correlation scores (higher than 0.9) between the topics recovered by our method and the ground truth, which show that our inference algorithm is fully capable of recovering latent topics from data.

Specifically, as shown in the figure below on the left, with increase in **the number of patients (number of documents)**, the correlation values increase which means that the model better learns the ground truth topic distribution. This is expected because there are more samples for the model to learn from. Notably, on a small corpus with only 1000 documents, our inference algorithm can still successfully recover topics (average correlation score higher than 0.92).

As shown in the figure below on the right, with increase in **the number of tokens (words per documents)**, the correlation values also increase This is also expected because again there are more samples for the model to learn from.

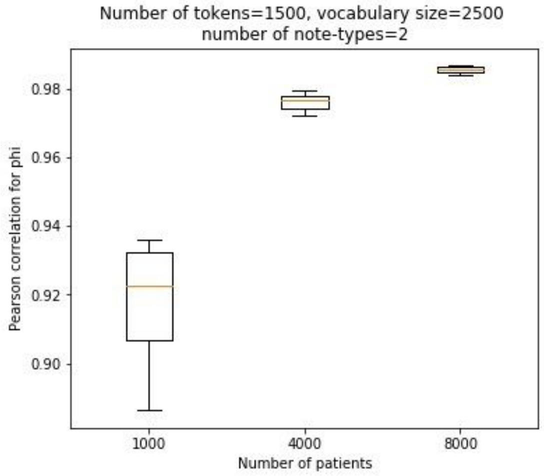

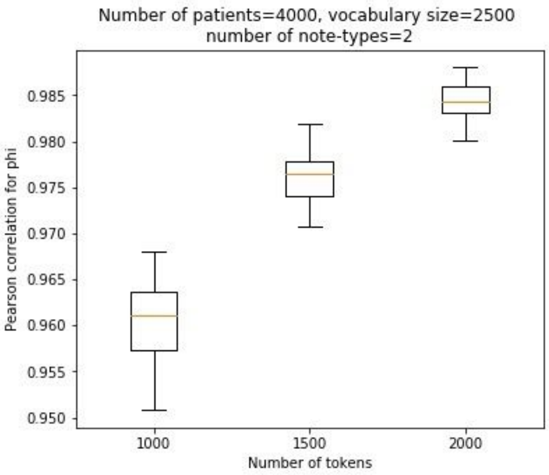

As shown in the figure below on the left, with increase in the **vocabulary size,** keeping the number of patients and number of tokens fixed, the correlation values decrease as the same number of samples are used to learn the topic distributions over a larger vocabulary. However, we note that the correlation values are still high (greater than 96%).

Similarly, as shown in the figure below on the right, with increase in **the number of note-types**, in order to **keep the overall number of tokens fixed**, we reduce the number of tokens from each note-type as a result of which, the correlation values decrease. Notably, even with 1500 tokens split in 4 note types, i.e. 375 tokens per note type, our inference algorithm can still successfully recover topics (average correlation score higher than 0.94).

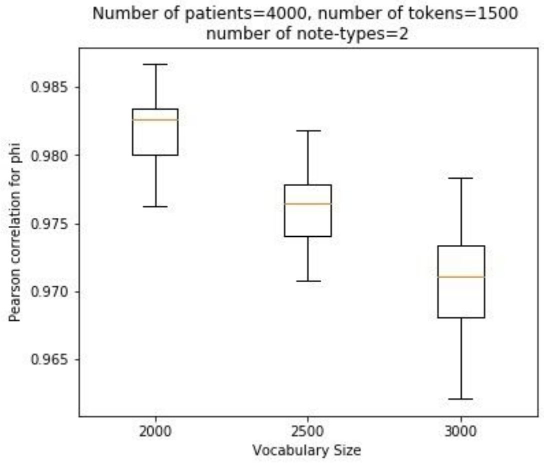

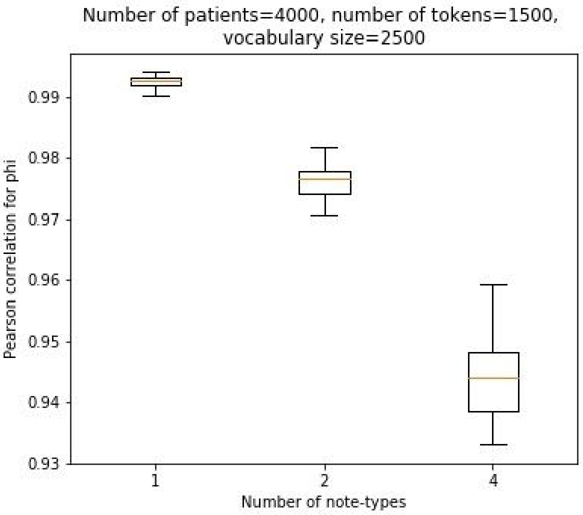

We also try modifying this experiment to the setting where **the number of tokens increases** (t times), and observe that the correlation values increase with increase in **number of note-types** (shown in the plot below). Here we note that the correlation values for t=1 and t=2 are lower because of the fewer number of tokens compared to the vocabulary size. As a result, the model has fewer samples to learn the topic distribution from.

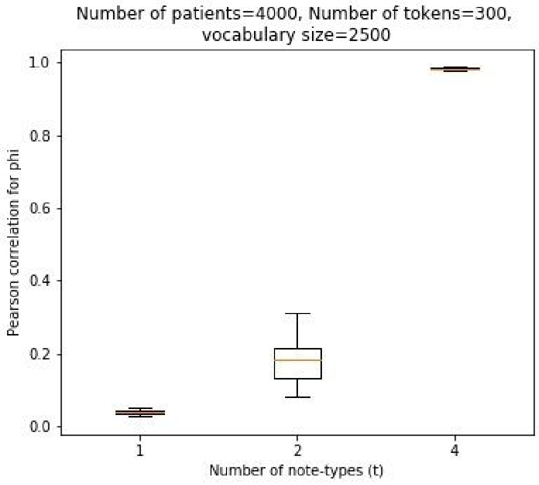

We conduct two more experiments where 1) we **drop a note type randomly** for each patient and measure the correlation between the learned topics and the ground truth topics and 2) we compare the correlation values between the patient-topic distribution (theta) of our method and a baseline by **varying the number of note types**. The baseline is an LDA method that considers the same words from different note types as the same word and simply adds up their frequency. For both these experiments, the number of patients, number of tokens and vocabulary size were fixed to 4000, 1500 and 2500. The results are in the two figures below. Even when some patients randomly miss 1 of the 2 note-types, the model does a good job of learning the ground truth topics as shown by the high correlation values. In the figure, when t={1, 2}, the box plot shows the correlation values between the learned and ground-truth topic distribution for note-types 1 and 2 and respectively. The multi-note Mixehr model better learns the patient-topic distribution (shown by higher correlation values) compared to the baseline LDA model that treats all note-types as the same.

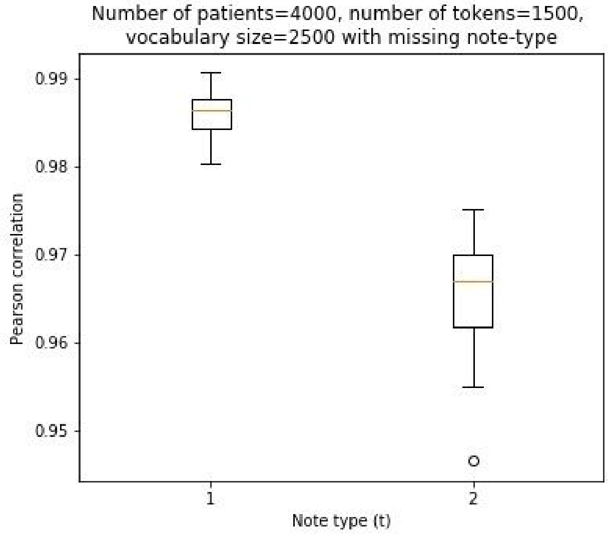

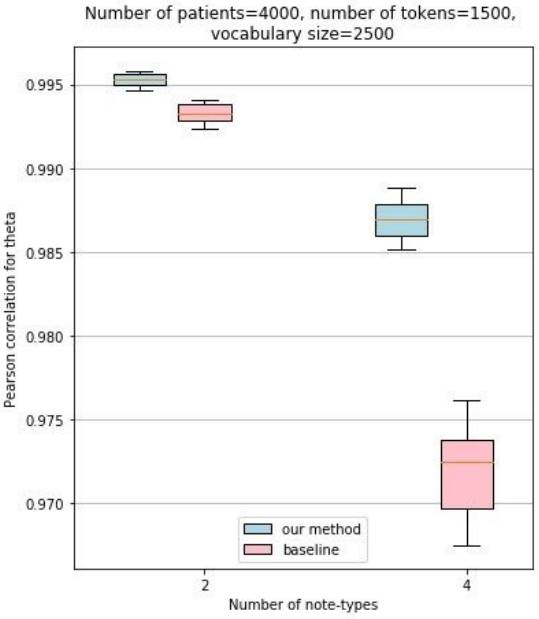

+ Page 5, 6: What predictors were used in the logistic regression models? The topic proportion estimates $\hat{\theta}_j$?

Our response: Yes the topic mixtures of documents are used for prediction, as stated in Section 3.1 (126-128).

+ Page 6: Why was the number of topics set to 50? Was this arbitrary or was this chosen by cross-validation on training data? The use of 50 topics could very likely contribute to overfitting especially given the large number of additional parameters being estimated in the proposed model. Since there is very little noticeable improvement in prediction quality, the data simply may not support a 50-topic model.

Our response: We experimented 10, 30, 50, 100 topics by measuring perplexity on the held-out documents and chose the best number of topics to go forward (Section 3.1, 115-117).

+ Page 6: The ROCs in Figure S2 do not support the claim that the "multi-note model achieved superior performance". The difference in AUROC is 86% vs. 85% and is presented without uncertainty estimates. Accounting for uncertainty, I would conjecture that the models are equivalent in prediction on this problem. This model does not, of course, have to be a major predictive breakthrough as its potential improvements to topic interpretability are interesting. However, I encourage the authors to (a) use less biased language when comparing the models' performance and (b) provide confidence intervals for the AUROC estimates throughout the paper.

Our response:

Thanks for the suggestion. We have tuned down the language, and we conducted additional experiments to show that there is little variation in our models' performance. Please see our response to the question "Was the impact of the pre-processing choices on model performance evaluated at all?".

+ Page 6, 7: Please define "cohesiveness". The claim that the "topics generated by the multi-note model had significantly more cohesiveness" is not supported unless I am missing something. This appears to be an entirely subjective claim.

Our Response:

a) We have clarified our definition of *cohesiveness* in the Section 2.3 (104-105)
b) Cohesiveness was necessarily measured qualitatively by two independent, blinded physicians. This process has been clarified in methods (Section 2.3, 106-109) and results (Section 3.2, 155-158).

+ Page 9: The claims in the last two sentences are overly strong given the evidence provided. It is not clear that the proposed model necessarily provides more meaningful topics (see comments elsewhere on this point). There is certainly limited or no evidence on this data set that the proposed model provides more accurate prediction of MV duration. As noted elsewhere, the differences in AUROC between the different models is negligible. Of course, this model *may* provide better predictive performance on other data sets. Indeed, as the authors hint at on p. 10, because the topics are not linked in the model to the outcome of interest as in, for example, supervised topic models (Blei & McAuliffe, 2008), there is no reason for the topic proportion estimates to be related at all to the outcome.

Our response:

The quality of topics is measured in terms of cohesiveness, which we have clarified in Methods (Section 2.3, 106-109) and Results (Section 3.2, 155-158), and our evaluation shows that in terms of cohesiveness the multi-note model we proposed generates topics of better quality.

+ Please provide code to reproduce the analyses described throughout the paper.

Our response: We have released the code in the revised version: https://github.com/li-lab-mcgill/heterogeneous_ehr

Minor Issues

+ Figure 1, p. 3: z_{ijk} and x_{ij} should have superscripts (t) to differentiate the different notes for patient j These superscripts appear in the distributional specification of both random variables on page 3.

Our response: Thank you for pointing out. We have fixed the notations.

+ Page 3, 4: References to the multinomial distribution should be replaced with the **categorical** distribution. This is also consistent with the distribution definitions for $z_{ij}^(t)$ and $x_{ij}^(t)$, which lack the normalization constants of a multinomial distribution but are consistent with the categorical distribution.

Our response: Thank you for pointing out. We have fixed the notations.

+ Page 5. Summary statistics of the document/note text should be provided. How long are these notes? How large is the vocabulary of each type of note?

Our response: Thank you for the suggestion. We have added summary statistics of the data in Section 2.2 (83-86): *Before preprocessing, the notes have 6091 words on average, and after preprocessing they have 301 words on average. The vocabulary for physicians' notes have 8948 words and the vocabulary for nursing notes have 8076 words.*

+ Page 5. Was the impact of the pre-processing choices on model performance evaluated at all?

Our response:

(Section S6 in supplementary material) As discussed in Section 2.2, our pre-processing procedures include standard practices for text processing and topic models, such as removing stop words, removing punctuations, and removing too frequent or infrequent words. Among all the operations, the design choice that might have a significant impact on our conclusions is the maximal document frequency for defining too frequent words, and therefore we studied the impact of this factor on the performance on the two prediction tasks. Specifically, on each task, we varied the maximal document frequency from 10% to 70% with an interval of 5%, and at each level we ran experiments 10 times with different random seeds for data splits.

Figures below show the average performance and error bar of the multi-note model on mortality prediction as measured by AUPRC and AUROC.

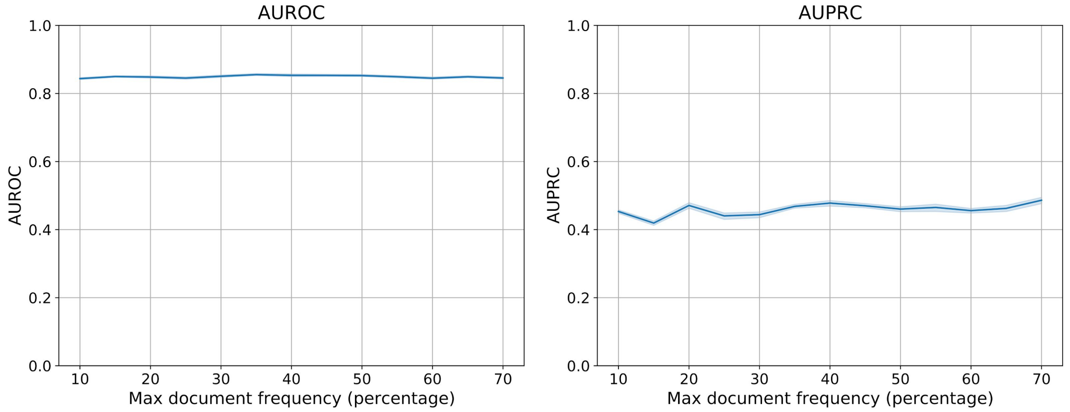

Similarly, figures below show the performance on PMV prediction.

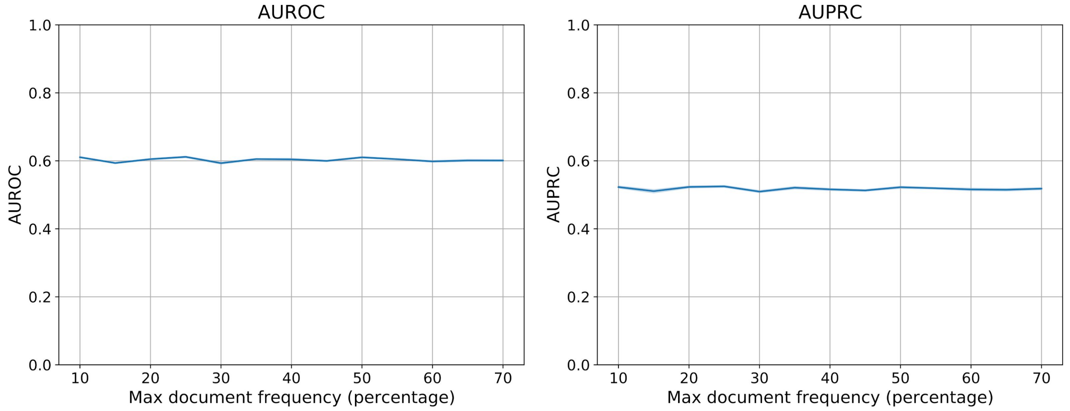

On both tasks, there is little variation in the performance across different random seeds, and the performance is consistent with varying maximal document frequency, indicating that our model is insensitive to the choice in data processing.

+ Page 5, 6: The outcome being predicted in the empirical application was dichotomized from a count variable (number of days), which can often reduce the information available and is not generally recommended. Please justify this decision or, perhaps better, use the original outcome.

Our Response:

Our primary outcome (prolonged mechanical ventilation defined as >7 days) [1, 2, 3] and secondary outcomes (in-hospital mortality) [4], were selected specifically because these outcomes are well supported in the literature and indeed are the conventional outcomes for early prediction scores. Both outcomes are clinically relevant, whereas a count in days is less appropriate for clinical decision making. Please see Introduction (22-27) and Section 2.2 (101-102) for detail.

+ Page 6 and elsewhere: Language used to compare the proposed model to the comparison models is overly optimistic and potentially misleading. A difference in AUROC of 66% vs. 65% is very small. Indeed, confidence intervals for the AUROC should be provided for all models as I suspect that they will indicate substantial uncertainty.

Our response: Please see our response to the question “Was the impact of the pre-processing choices on model performance evaluated at all?”.

+ Page 7: What do the numbers in parentheses for the average interpretability scores represent? Standard deviations? Confidence limits? On a related note, if the ratings are between 1 and 5, a rating of 4.22 + 1.15 = 5.37 is impossible. Finally, a direct test comparing the interpretability rating of the rater(s) would be useful to avoid overinterpreting what appear to be quite noisy estimates of interpretability. Accounting for uncertainty, there may be no statistically significant difference in interpretability between the two models.

Our response: Thanks for pointing out. We have clarified that they are standard deviations (Section 3.2, 159-160).

+ Page 8: What measure of correlation was used? Pearson's correlation (the usual default) is inappropriate for correlating mixture proportion estimates with a duration variable because of the range restriction on the mixture proportion estimates. A better option would be Spearman's or Kendall's correlation coefficients.

Our response: We chose Pearson’s correlation coefficient because it is a normalized metric whose magnitude reflects the strength of linear correlation, in the range of -1 to 1, and the range restriction of the variables has no impact on the correlation. We also tried Spearman’s and Kendall’s correlation coefficients and observed similar results (Section 3.3, 168-171).

+ Figure 4, Page 9: Please describe the components of this boxplot as different graphing software uses different conventions. Are the whiskers quartiles? Confidence limits? What do the bounds of the box indicate? What are the points indicating and are they overplotted (jitter the points if so)?

Our response: Thank you for pointing out. We have clarified the boxplot components in Figure 4's caption: *The horizontal lines in the box represent the median and the box represent the range between 25% and 75% quartile of the data.*

Typos

+ Page 4: After Equation (3), please remove the fragment "The hyperparameters α_k and β_{tk}".

Our response: Thank you for pointing out. We fixed the typo.

Reviewer 3

The authors introduced a novel hierarchical topic model method for clinical notes retrieved from Harvard teaching hospitals. The method and its interpretation are interesting from both machine learning/NLP and clinical informatic perspective. I have following comments

1. The topic modelling method, by its nature, belongs to unsupervised distributional embedding learning, which is a traditional branch of text embedding widely used. In recently years, the clinical NLP community has been dominant by "BERTologists" (the group of researchers focusing on transformer based model). Could the authors clarify the different use cases and advantages of method present in this manuscript compared with transformer based models?

Our response: Thank you for raising this question. The model used in this study has several advantages over Transformer-based models. First, our model is more computationally efficient compared to Transformer-based models. Our experiments were all conducted on CPU machines, whereas Transformer-based models usually require GPU acceleration. This is especially important for deployment in healthcare settings which might not be optimized for heavy computations. Second, our model offers a higher level of interpretability by assigning documents with semantic topics' distributions and by defining topics as distributions over words. Indeed, in our study we evaluated the interpretability of the model in terms of both documents' topic mixtures (Section 3.3) and the learned topic distributions (Section 3.2). In contrast, Transformer-based models represent data in latent space which is not explicitly meaningful, and the hidden activities within their building blocks are not explicitly meaningful either. Third, as a result of model components being interpretable, our model's hyperparameters are also more meaningful (e.g. the number of topics), and thus allowing for more evidence and reason-based hyperparameter configurations.

2. One interesting feature found in this method is topic discovery and interpretability of the model. I would suggest the author reflect these points in abstract and even the title for readers without much machine learning backgrounds.

Our response: Thank you for the suggestion. We have revised the abstract and title for better communication.

3. When predicting mortality, models present in this manuscript has AUCROC<0.70. As MIMIC3 dataset is from ICU and many publications claim AUCROC >0.80, it is necessary for the authors to justify this relatively low performance. If the focus is not performance, it should be clearly mentioned early in the text.

Our response: Thank you for the question and suggestion. Indeed, the main advantage of this model is not its predictive power. We have revised the text to highlight the focus of this study. However, we still argue that our model has a strong predictive power. In particular, MIMIC3 includes many types of data other than medical notes (e.g. diagnosis codes, lab tests, prescription codes), and it is reasonable to expect using these additional information would lead to better predictions. Also, as detailed in Section 2.2, we applied careful data filtering before conducting experiments to ensure rigorousness, which excluded the majority of samples in MIMIC3, including the samples that might make predictions trivial. The reduced data size and the exclusion of "easy" samples also affect the final AUROC scores.

References:

[1] Systematic review and meta-analysis of studies of the timing of tracheostomy in adult patients undergoing artificial ventilation. *Griffiths J, Barber VS, Morgan L, Young JD BMJ. 2005 May 28; 330(7502):1243.*

[2] Boles JM, Bion J, Connors A, Herridge M, Marsh B, Melot C, Pearl R, Silverman H, Stanchina M, Vieillard-Baron A, Welte T Eur Respir J. 2007 May; 29(5):1033-56.

[3] Federico Pappalardo, Annalisa Franco, Giovanni Landoni, Paola Cardano, Alberto Zangrillo, Ottavio Alfieri, Long-term outcome and quality of life of patients requiring prolonged mechanical ventilation after cardiac surgery, *European Journal of Cardio-Thoracic Surgery*, Volume 25, Issue 4, April 2004, Pages 548–552

[4] Vincent JL, Moreno RP. Clinical review: Scoring systems in the critically ill. Crit Care. 2010;14(2):142–311
