# Supplementary material for: Mining heterogeneous clinical notes by multi-modal latent topic model
Source: PLoS One. 2021 Apr 8;16(4):e0249622. doi: 10.1371/journal.pone.0249622 (PMC8031429; doi:10.1371/journal.pone.0249622)
Supplement: Attachment — Submitted filename: Response to Reviewers.pdf [file pone.0249622.s003.pdf]

Response to Reviewers

Reviewer 2

Summary

I thank the authors for addressing most of the questions and comments I had from the first version of their manuscript. In particular, I applaud them for conducting and reporting a simulation study to evaluate the performance of their estimation algorithm. This provides valuable information about the proposed algorithm's ability to successfully estimate the proposed model, particularly for data sets with similar characteristics. I also thank the authors for providing access to the code for the proposed algorithm and its application via Github. I have several remaining points for the authors to consider, which I list below.

Interpretability

+ Page 7, Line 159-160: As I mentioned in my review of the original manuscript, a direct test comparing the interpretability rating of the rater(s) would be useful to directly test the difference in interpretability ratings between the topics from the proposed model and the single-note model. I did so using paired t-tests (under a range of assumed correlations since I don't have the original scores) and find that the difference is statistically significant across different potential correlations, so I agree that there is improvement in physician rating of interpretability. My main suggestion here is to report such a test directly in addition to the means and standard deviations to provide clear evidence of the claim that is highlighted in both the abstract and in the Discussion (Line 190-193) that interpretability may be better using the multimodal topic model instead of the standard LDA model in this example.

Our response: Thank you for the suggestion. We conducted a two-sided t-test between the physician ratings of the multi-note topic model and the single-note topic model (i.e., the standard LDA model) in R and obtained a p-value equal to 0.001298 (output pasted below). This indicates that the difference between these two models in terms of physician's ratings is statistically significant. We added this observation into the manuscript at **lines 177-179 on page 8**.

```
> t.test(subset(df, method=="single-note")$score, subset(df, method=="multi-note")$score)

        Welch Two Sample t-test

data:  subset(df, method == "single-note")$score and subset(df, method == "multi-note")$score
t = -3.3121, df = 98, p-value = 0.001298

alternative hypothesis: true difference in means is not equal to 0

95 percent confidence interval:
 -1.215361 -0.304639
```

sample estimates:
mean of x mean of y
3.46 4.22

Prediction

+ Page 5, Line 131-132: As I mentioned in my review of the original manuscript, the ROCs in Figure 2 and in Figure S2 do not support the claim here that the "multi-note model performed better compared to single-note models". For example, the difference in AUROC from Figure S2 when predicting whether a patient will be on a mechanical ventilator for more than 7 days or not is 86.0% vs. 85.2%, an arguably negligible difference, which is presented without uncertainty estimates such as the standard deviation across the 5-fold cross-validation. Accounting for uncertainty, I would conjecture that the models are equivalent in prediction on this problem. Again, I encourage the authors to provide confidence intervals for the AUROC estimates throughout the paper (e.g., via bootstrapping as in the pROC R software package; Robin et al., 2011, doi: 10.1186/1471-2105-12-77).

Our response: We thank the reviewer for the suggestion. Following this suggestion, on both prolonged mechanical ventilation (PMV) prediction and mortality prediction, we conducted experiments by splitting the data into 5 folds randomly for 10 different splitting seeds. In each experiment, we performed a 5-fold CV to get AUROC and AUPRC. Then we calculated the average and standard deviation of the AUROC/AUPRC over the 10 experiments. These results are included in **Figure 2** in the main text (**Figure R1**) and Figure S2 in Supplementary (**Figure R2**), and the statistical significance of the difference between methods is discussed in **lines 110-126 on page 5 and lines 134-142 on page 6**.

Overall, the standard deviation over the 10 experiments are quite small for each method. On PMV prediction, in particular, the multi-type model achieves AUROC equal to 0.668 with standard deviation (std) equal to 0.008. Hence, the 95% confidence interval (CI) is [0.668 - 1.96 * 0.008/10^0.5, 0.668 + 1.96 * 0.008/10^0.5] = [0.6630, 0.6730]. The best single-note type model (diff-word) achieves on average 0.660 +/- 0.008 std (i.e., [0.6550, 0.6650] 95% CI). Therefore, the AUROC of the multi-note model is higher than the best single-note model but the difference is *not* statistically significant at 95% CI. However, AUROC tends to be insensitive to unbalanced data. We therefore turned to AUPRC.

In terms of AUPRC, the multi-note model achieves on average 0.408 +/- 0.007 (std), while the best single-note model achieves on average 0.392 +/- 0.008 (std), and the 95% confidence interval in terms of AUPRC are [0.404, 0.412] and [0.387, 0.397], respectively. This shows that the AUPRC of the multi-note model is significantly higher than the AUPRC of the best single-note model at 95% CI.

On mortality prediction, the multi-note model achieves on average 0.861 +/- 0.004 (std) in terms of AUROC and [0.859, 0.863] 95% CI. The best single-note (same-word) model achieves on average 0.845 +/- 0.004 (std) and [0.843, 0.847] 95% CI. In terms of AUPRC, the multi-note model achieves on average 0.419 +/- 0.011 (std) and [0.412, 0.426] 95% CI, while the single-note model achieves on average 0.404 +/- 0.008 (std) and [0.399, 0.409] 95% CI. These indicate that both the AUPRC and AUROC of the multi-note model are significantly higher than those of the best single-note model at 95% CI.

In summary, we observe that consistently higher AUROC and AUPRC from the multi-note model compared to the single-note models in terms of PMV and mortality prediction. And the improvements of multi-note over single-note are statistically significant. Therefore, we can conclude that while the margin of difference can be small, the multi-note model is significantly better than the single-note models on both prediction tasks.

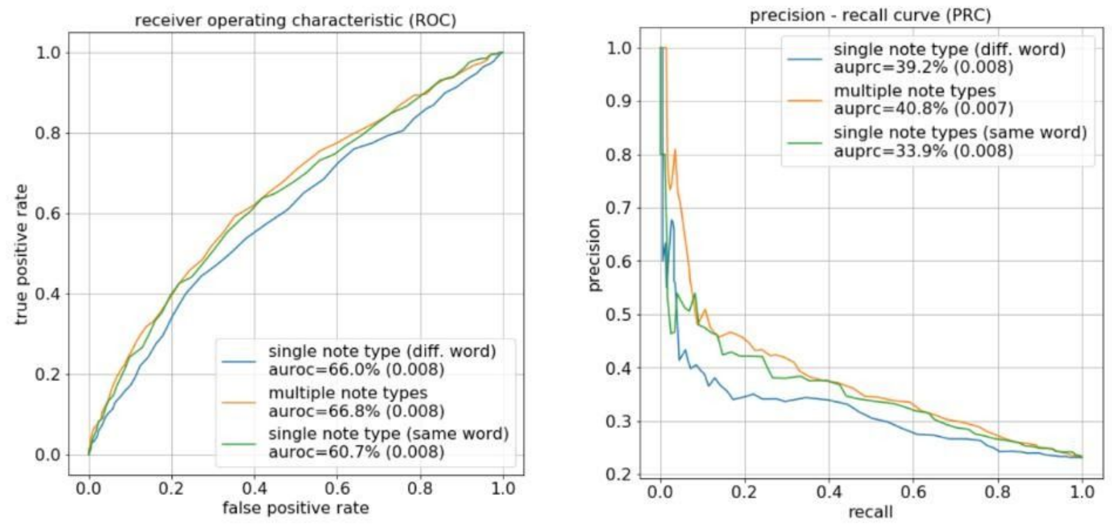

Figure R1 (Figure 2 in the main text). ROC and Precision-recall curve for binary prolonged mechanical ventilation (PMV) prediction. We trained the two single-note topic models and the multi-note topic models on the first 48 hours of the clinical notes for each patient. We then trained a separate logistic regression classifier that took the patient-note topic mixture as input and predicted whether the patient is going to stay on MV for more than 7 days. The trained topic models and logistic classifiers were then applied to the test patients to make the predictions of PMV duration. The prediction accuracy was evaluated by ROC and precision-recall curves. The figure inset shows the AUROC and AUPRC values for each model, and the standard deviations across 10 random splits are in parenthesis.

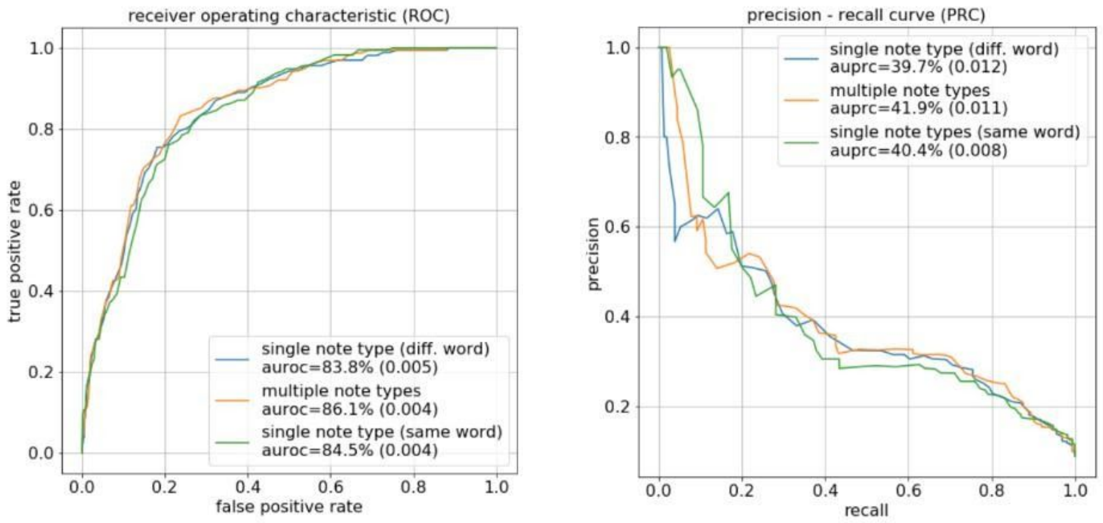

Figure R2 (Figure S2 in Supplementary Information). ROC and Precision-recall curve for mortality prediction prediction.

+ As I mentioned in my original review and in the first bullet point above, claims in the results sections and discussion (e.g., Lines 130-132, 193-194) are not supported by the evidence provided: The authors claim that the proposed model "performed better compared to single-note models, as measured by AUROC and AUPRC" and "our model confers more accurate prediction of duration of MV". However, the AUROC and AUPRC values are, as I mentioned above, quite similar. There is no strong statistical evidence (unless confidence intervals for each AUROC/AUPRC value per model and for the difference in AUROC/AUPRC between models are provided) that prediction performance differed between the two models. I would echo comments from other reviewers that even if these AUROC values differ significantly from a purely statistical inference perspective, that the very small magnitude of such a difference needs to be defended. Alternatively, the authors could omit claims that their model's predictive performance is better than single-note models and focus on the improved interpretability of the topics extracted by their model. To clarify, I do not believe that they need to show better predictive performance for this paper to be a valuable contribution to the literature. However, I do not want their claims in the results and discussion to potentially misread a casual reader.

Our response: We thank the reviewer for the advice. As detailed in our response to the point above, we have conducted additional experiments to confirm that the differences between the models are mostly statistically significant although the absolute difference can be small. We completely agree that we should have been more cautious in making these claims to not misguide readers. We have revised the manuscript accordingly (**line 134 on page 6**, **line 145 on page 6** and **line 213 on page 9**).

Errata

While the overall conclusions and results in the paper are clear, the paper would benefit from careful proofreading for minor typographical and grammatical errors (e.g., definite and indefinite articles such as "a" and "the" are omitted throughout the paper) before publication.

All of the authors have carefully read through the manuscript and fixed all of the typographical and grammatical errors throughout the paper.
